# Selforganization of modular activity of grid cells

**DOI:** 10.1002/hipo.22765

**Published:** 2017-08-14

**Authors:** Eugenio Urdapilleta, Bailu Si, Alessandro Treves

**Affiliations:** ^1^ División de Física Estadística e Interdisciplinaria Centro Atómico Bariloche S. C. de Bariloche Río Negro 8400 Argentina; ^2^ Shenyang Institute of Automation, Chinese Academy of Sciences Shenyang 110016 China; ^3^ Cognitive Neuroscience, SISSA Trieste 34136 Italy

**Keywords:** grid cells, modules, self‐organization

## Abstract

A unique topographical representation of space is found in the concerted activity of grid cells in the rodent medial entorhinal cortex. Many among the principal cells in this region exhibit a hexagonal firing pattern, in which each cell expresses its own set of place fields (spatial phases) at the vertices of a triangular grid, the spacing and orientation of which are typically shared with neighboring cells. Grid spacing, in particular, has been found to increase along the dorso‐ventral axis of the entorhinal cortex but in discrete steps, that is, with a modular structure. In this study, we show that such a modular activity may result from the self‐organization of interacting units, which individually would not show discrete but rather continuously varying grid spacing. Within our “adaptation” network model, the effect of a continuously varying time constant, which determines grid spacing in the isolated cell model, is modulated by recurrent collateral connections, which tend to produce a few subnetworks, akin to magnetic domains, each with its own grid spacing. In agreement with experimental evidence, the modular structure is tightly defined by grid spacing, but also involves grid orientation and distortion, due to interactions across modules. Thus, our study sheds light onto a possible mechanism, other than simply assuming separate networks a priori, underlying the formation of modular grid representations.

## 1 INTRODUCTION

An accurate representation of their own position in space is thought to be a fundamental requirement for animals to perform most of their activities, which entail moving around in the environment. The spatial cognitive map theory outlines such a flexible representation (Tolman, 1948), in which an animal creates schemas that incorporate spatial knowledge. For years, the cognitive map has been associated with the hippocampus (O'Keefe & Nadel, 1978), where, in rodents, principal cells have been seen to represent specific locations in paradigms that require the animal to move in a given environment (O'Keefe & Dostrovsky, 1971). In 2005, the discovery of grid cells, one synapse upstream to the hippocampus, in the medial entorhinal cortex, appeared to reveal a much more geometrical, rigid implementation of the cognitive map concept (Hafting, Fyhn, Molden, Moser, & Moser, 2005; Moser, Kropff, & Moser, 2008). In small flat environments, these grid cells fire at multiple locations with a regular hexagonal structure; unlike place cells, they fire in all environments and maintain rigid coactivity relations (Fyhn, Hafting, Treves, Moser, & Moser, 2007). Reminiscent of a stack of sheets of millimeter paper stapled together, nearby cells display the same hexagonal pattern, although phase‐shifted, in one environment and across environments. At the same time, grid spacing increases gradually from the dorsal to the ventral end of entorhinal cortex, suggesting that the same representation is replicated at multiple scales (Brun et al., 2008). At a given scale, the observed rigidity, that is, that phase relationships between nearby grid cells are preserved during remapping (Fyhn et al., 2007), makes one think of strong local networks, which have been hypothesized to approximate a metric system (Moser & Moser, 2008).

Do these parallel representations occur at any possible scale? By systematic analysis of multiple unit recordings in single animals, grid cell activity was found to be organized in discrete modules, with only four to five spatial scales observed in each animal (Stensola et al., 2012). A simple explanation of such modular organization is that it is pre‐wired: grid cells may emerge already affiliated with one of four to five networks, within which units interact strongly, while they interact weakly, if at all, across networks. A model that offers such an account, where units learn their affiliation among different pre‐defined scales, has already been presented (Pilly & Grossberg, 2014). We want to assess the alternative hypothesis that the modular structure is not pre‐wired, and instead self‐organizes. Our approach is based on a model, the self‐organization model previously proposed for the formation of grid cell activity (Kropff & Treves, 2008). Here, we consider the concomitant self‐organization of the modular structure for an extended network of grid units.

## 2 THE MODEL

In the model, three ingredients are necessary for the development of a coherent grid pattern. First, a cellular adaptation mechanism suppresses firing within a certain time window after spells of intense firing; for a rodent moving in an environment at a certain average speed, and single units responding primarily to location‐specific inputs, this tends to suppress firing in a ring around each region where the inputs happen to be stronger. In other words, adaptation acts indirectly as a spatial band‐pass filtering. Second, the self‐organization of multiple fields into the hexagonal pattern requires the gradual reinforcement of those initially random fields that happen not to suppress each other. This can be achieved by Hebbian plasticity on the afferent connections to a single unit, and slowly produces hexagonal grids at the single unit level, with random orientations. Third, different grid units endowed with the same adaptation time constants produce, in a flat environment, distinct but aligned hexagonal patterns. The addition of collateral interactions, modulated by the direction selectivity of each unit (Kropff & Treves 2008), has been shown to have the capability to align the grid patterns into a common orientation for groups of units (“modules”), an alignment which is preserved during remapping (Si, Kropff & Treves, 2012). Strictly speaking, this network model accounts for the conjunctive (grid x head direction) activity found in layer III and deeper layers of mEC (Sargolini et al., 2006); however, it can be extended to include also, as a downstream population of units, those expressing pure grid activity, as observed in layer II, without any head direction modulation (Si & Treves, 2013). In the model, the information about the animal's position may be delivered by different sources, including a major feedback projection from CA1 to deep layers in mEC (Bonnevie et al., 2013; Moser et al., 2014). As the self‐organization character of the model requires Hebbian learning to refine and reinforce a specific lay‐out of the multiple fields of each unit, it depends on input statistics, whether from CA1 or elsewhere, and ultimately on the exploration of the environment itself. This is what makes the model predict different types of lay‐out as the geometry or the dimensionality change, in comparison to a simple planar Euclidean geometry (Stella, Si, Kropff, & Treves, 2013; Urdapilleta, Troiani, Stella, & Treves, 2015; Stella & Treves, 2015). As previously discussed (Si & Treves, 2013), the dependence of the model on learning conditions may be critical particularly during development. In this sense, the timescale to develop a population‐based grid structure in the grid or conjunctive layer is in the order of days of active exploration. Detailed information about the model is given in the Appendix.

### 2.1 Prewired modular organization

**Figure 1:**
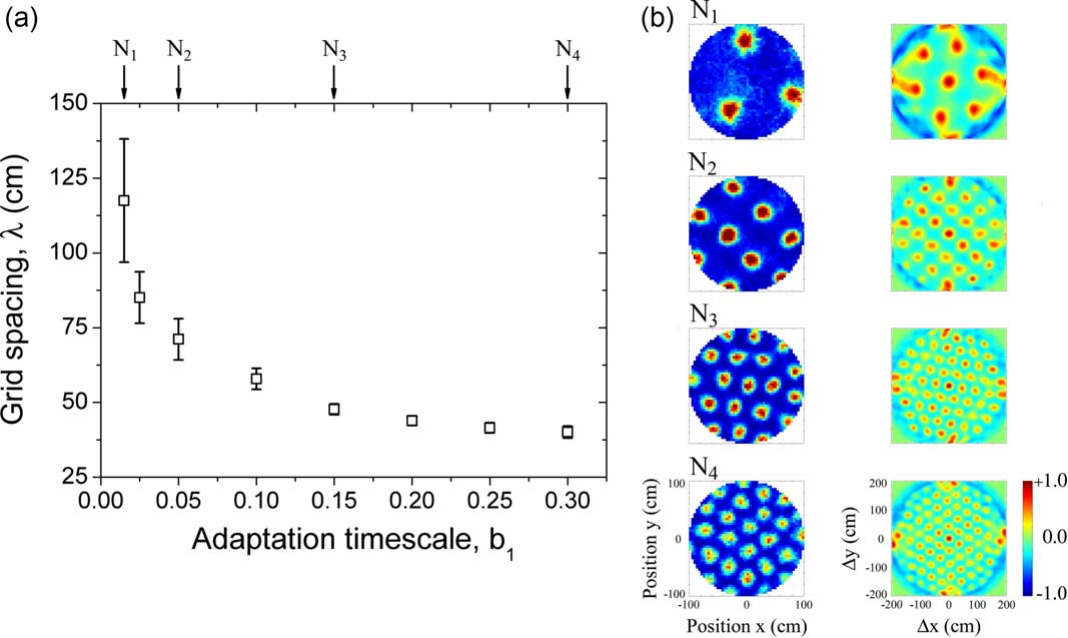
Homogeneous networks with different grid spacing. (a) The parameter b_1_ in the self‐organization model largely controls this property. Networks with large values of *b*_1_ are tightly organized in a common representation with small grid spacing, whereas networks with a small value of *b*_1_ express a less coherent representation with large grid spacing and more variability. (b) Representative examples of single unit activity from homogeneous ensembles, as indicated in panel (a) (different N_i_). Left panel: Spatial map. Right panel: Auto‐correlogram. Axis and labels are indicated in the lower panels [Color figure can be viewed at wileyonlinelibrary.com]

In a simple scenario, the self‐organization of different modules may reduce to the parallel self‐organization of largely separate networks internally homogeneous in terms of the single‐unit properties, including grid spacing. A common orientation is produced by collateral interactions within the units of the module, without, or with weak projection to other modules. In Figure 1, we show the simulated emergence of typical grid representations in homogeneous networks, each assigned specific adaptation properties (here restricted to the manipulation of the b_1_ inverse time constant). The parameter b_1_, which in the model sets the timescale of the adaptation process, controls the grid spacing developed by the units (see Figure 1a). A common orientation for all units in the ensemble and diverse phase relationships between them are organized by the collaterals and by individual head direction modulation. When isolated units are simulated with directional modulation, there is a slow temporal drift of the firing map along the opposite direction of the associated head‐direction selectivity. This is a consequence of the adaptation properties (modeling fatigue), exerting a larger effect in a given direction for each conjunctive unit. The effect is essentially the same as the one reported for the experience‐dependent properties of the firing fields in place cells (Mehta, Barnes & McNaughton, 1997; Mehta, Quirk & Wilson, 2000). When conjunctive units interact with each other, drift is reduced because neighboring units “push” each other along all directions, eventually establishing a kind of strained or frustrated fixation, except when the directional modulation is very strong. For units developing a larger grid spacing (small b_1_), individual fields are also larger and the drift effect lasts longer. To compensate this (assuming it makes sense for the different modules to reach stability at about the same time), as the grid spacing increases, the head‐directional modulation should be less tuned. This weaker modulation at large grid spacing has an impact on the variability observed in the geometrical properties of the pattern, which generally display a looser overall alignment. This variability, both in grid spacing and in alignment, is also observed in experimental studies (Stensola et al., 2012). The (putative) computational requirement of variable head direction modulation strength exactly matches what was recently found about the topographical organization of head direction selectivity along the dorso‐ventral axis in the mEC (Giocomo et al., 2014). Representative examples of single‐unit grid activity displayed by the parallel development of networks are shown in Figure 1b, as well as the prototypical auto‐correlograms used to characterize grid patterns. As expected, therefore, the parallel development of noninteracting networks successfully explains modular formation, by relying on pre‐wired compartments.

### 2.2 Modular self‐organization

**Figure 2:**
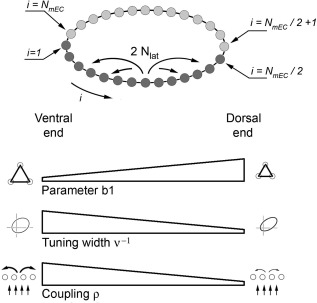
Heterogeneous network of conjunctive grid units. Upper figure: To avoid boundary effects, the network is arranged as a ring, with individual unit properties linearly changing from dorsal to ventral end, along either half‐ring. Lower panels: variable properties are: (a) the parameter *b*_1_, determining the grid spacing of individual firing maps, (b) the inverse tuning width ν, quantifying head‐direction modulation, (c) the coupling ρ, weighting collateral to feedforward connectivity

As an alternative, we consider the hypothesis that the self‐organization of the modules may occur by a spontaneous breaking of a continuous distribution of time constants in a single heterogeneous network, via interactions that produce discrete domains of grid spacing. This can be considered analogous, at the level of firing maps, to the synchronization of interacting oscillators exhibited by the Kuramoto model (Acebron, Bonilla, Perez Vicente, Ritort, & Spigler, 2005; Kuramoto, 1984). In that case, due to the interactions, elements with different natural frequencies acquire a common rotation, if the coupling is strong enough. In our model, instead, we consider a smooth gradient in the parameter *b*_1_, possibly accompanied by other variable cellular properties along the dorso‐ventral axis; and collateral connections that provide for interactions within a given range along the same axis. As shown in Figure 1, each specific value of *b*_1_ in isolation (or in an ensemble of identical elements) would imply the emergence of a specific value of grid spacing. Given a population of *N*_mEC_ units, labeled by *i* (*i* = 1, …, *N*_mEC_), to avoid boundary effects, we find it convenient to take the index *i* to run along the axis twice from the ventral to the dorsal and back to the ventral end (Figure 2). Then units lie effectively on a ring, and each interacts via collateral connections with *N*_lat_ other neighboring units on each side. To simplify, interactions are dependent on distance along this unidimensional circular axis, and thus interactions across the two branches joining ventral to dorsal ends are not considered. We further assume, in our model, that the “natural” grid spacing λ (i.e., the grid spacing developed in isolation or in an ensemble of identical units) should be linearly related to the distance Δ from the dorsal end of mEC (this is a conservative assumption, as will be clear below). Since one can characterize the relationship between the grid spacing, λ, and the parameter *b*_1_ as, roughly,

(1) $$\lambda \left(b_{1}\right)\;=\;a_{0}+\;a_{1}\;\text{exp}\left[-b_{1}/B_{1}\right]$$

valid for the entire range except possibly very large values of λ (see Figure 1), by setting the value *b*_1_ for individual units in the heterogeneous population as

(2) $$b_{1}\left(i\right)\;=\;B_{1}\;\text{ln}[c_{1}/(\Delta -c_{0})]$$

with appropriate values *c*_0_ and *c*_1_ and *B*_1_ (obtained, e.g., via the evaluation of selected values of *b*_1_ at the dorsal/ventral ends to approximately match experimental measures) one recovers the desired linear relation between Δ and λ. In particular, we set *B*_1_ from Figure 1 as 0.080 [*t* ^−1^] (in inverse arbitrary time units). Concomitantly, inspired by (Giocomo et al., 2014), we impose progressively sharper head‐direction selectivity towards the dorsal end, and also decreasing collateral‐to‐feedforward weights. In detail,

(3) $$\text{Inverse}\;\text{of}\;\text{the}\;\text{width}\;\text{of}\;\text{the}\;\text{tuning}\;\text{function :}\nu \left(b_{1}\right)\;=\;d_{0}+\;d_{1}\;\text{exp}\;\left(-b_{1}/B_{1}\right)$$

(4) $$\text{Strength}\;\text{of}\;\text{collaterals}:\rho \left(b_{1}\right)\;=\;e_{0}+\;e_{1}\text{exp}\left(-b_{1}/B_{1}\right)$$

Parameters *d*_0_ and *d*_1_ are defined in order to obtain ν (dorsal) = 1.0 (sharply tuned head direction selectivity) and ν (ventral) = 0.2 (slightly tuned), and *e*_0_ and *e*_1_ to obtain ρ (dorsal) = 0.2 (weak influence of collaterals), and ρ (ventral) = 0.4 (stronger influence).

In addition to endowing units with these variable properties along the model ventro‐dorsal axis, other aspects of the model are: (1) To reduce confounding effects, inputs from all place cells are taken to reach each conjunctive unit, so any scale variation in place fields and topography in CA1‐to‐mEC connections is disregarded, (2) Collateral interactions are localized to the neighborhood of each unit (|*i* − *j*| = 1  … , *N*_lat_), and are sparse, (3) To avoid boundary effects at dorsal/ventral ends, they are joined together, but via the ring network mentioned above; in practice, this creates a mirror image of the dorso‐ventral axis, with the properties defined above selected according to the dorso‐ventral position along either half of the ring, see Figure 2.

“Gradient smoothness” is controlled by the ratio *N*_lat_/*N*_mEC_. By running distinct simulations, all with the same extreme values for the variable properties, and all with the same *N*_lat_ (2*N*_lat_ = 100), but with an increasing number of units in the conjunctive grid layer, *N*_mEC_, we tested different gradients. In particular, in an all‐to‐all (but sparse) connectivity pattern, we did not observe the emergence of modules, although a mild effect of aggregation between close units was observed, interleaved with the natural development of the intrinsic grid spacing, given by the individual values of b_1_ (not shown). As expected, this aggregation process was strengthened as *N*_mEC_ increases. The first signs of clear modularity were observed for *N*_mEC_ = 600, the results reported hereafter.

## 3 RESULTS

**Figure 3:**
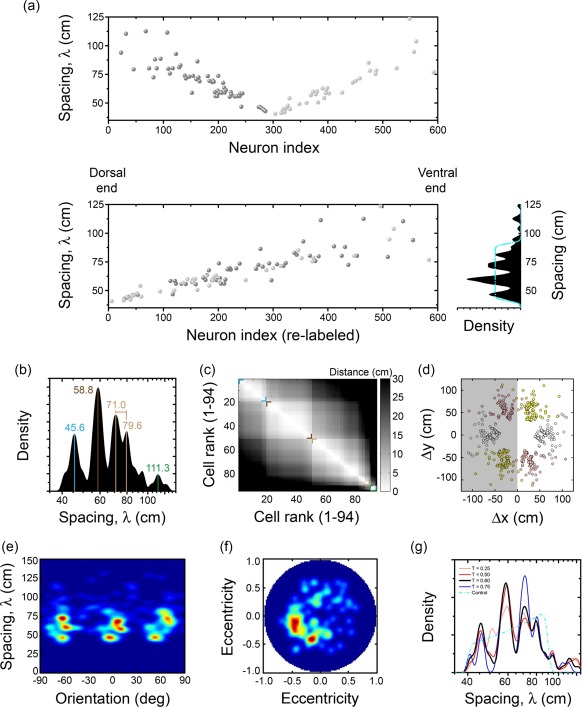
Self‐organized modular development of grid activity. (a) Left: Spacing displayed by individual units, labeled and colored according to Figure 2 (top), and relabeled dorsal‐to‐ventral (bottom). Right: Grid spacing histogram. By construction (see main text), a noninteracting population would produce a flat distribution (cyan dash‐dotted line). (b) Grid spacing histogram in semi‐logarithmic scale. Modules are indicated by colored vertical lines, which mark clear relative maxima. The spacing in the third module ranges between the values indicated in light brown. (c) Distance between units ranked according to their grid spacing. Modules are indicated by colored corners. (d) Centers of the surrounding fields closest to the origin in the autocorrelogram. Points on the negative semi‐plane (shadowed) are a π‐rotated version of those on the positive one. (e) Joint histogram of spacing × orientation. (f) Distortion map (see text below). (g) Grid spacing histogram in semi‐logarithmic scale, for units selected with different thresholds (indicated in the inset). In addition, the control distribution (uniform density in linear scale) is superimposed [Color figure can be viewed at wileyonlinelibrary.com]

The self‐organization of modular activity in a heterogeneous network of interacting conjunctive units is evident from the clustering of individual grid patterns in a few common representations. Grid patterns were identified through standard procedures, based on spatial autocorrelograms (Hafting et al., 2005; Sargolini et al., 2006; Stensola et al., 2012). In brief, units with grid scores above a chosen threshold (see Figure 3) were selected for subsequent analysis. From the spatial autocorrelograms, we identified the six fields closest to the origin and selected three as the “axes” of the grid representation. Following (Stensola et al., 2012), only units surpassing fixed criteria on the inter‐axis angle and the distortion between individual grid distances (distances from the origin to the respective fields) were further analyzed. After this filter, the grid spacing of each grid unit was measured as the arithmetic mean of grid distances along the three axes. As shown in Figure 3a (top panel), such spacing follows the overall trend expected from the setting of the parameter *b*_1_, from the ventral to the dorsal and back to the ventral end. When units are relabeled progressively according to their dorso‐ventral position_,_ the same trend is conserved but a step‐like behavior is revealed, with a small number of steps (left bottom panel). This is clearly shown by the multi‐peaked distribution of grid distances (right panel), which is different from the gradual distribution designed before adding interactions. We can also observe that between the steps (left panel), there are gaps along the horizontal dimension. These results from units that do not pass the criteria for further analysis and are therefore not shown, because they do not express a clear spacing. This effect is reduced by lowering the threshold on grid score. Overall, this means that units relax grid quality between clearly delimited modules (but, as we demonstrate in Figure 3g, the very same modules are still present even with units with very poor grid characteristics). Interestingly, Figure 3a shows that the clustering in grid spacing values develops similarly along both branches of the dorso‐ventral gradient, indicating that it is a robust effect.

In agreement to Stensola et al. (2012), as the spacing in each module increases, its dispersion increases as well. To visually minimize this effect, a representation of the distribution of grid spacing in semi‐logarithmic scale is convenient. As shown in Figure 3b, in this simulation four modules are clearly visible (indicated by different colors), although some peculiarities are worth mentioning. First, the third module (light brown in the figure) is associated to two peaks, very close in magnitude. This is likely a feature of this particular simulation and it may be associated to finite size and finite time effects. However, as we point out below, a stronger collateral effect and, perhaps, a softer gradient in individual properties may be necessary to resolve modules, particularly at larger scales. Second, some units are associated to the tail of this third module, but more likely they are isolated units (see Figure 3a, left bottom panel, and Figure 3e). Both observations suggest that a stronger collateral interaction at this particular topographical location, given by ρ in our model, may be necessary to coalesce the aggregated units. Third, the fourth module is scarcely populated, in agreement to experiments to date. In fact, whatever the criterion for the inclusion of units in the grid category, fewer units pass it close to the ventral end. This may be an indication that grid networks at larger scales are less stable, and that the experimental evidence for an approximately geometric scaling of the spacing (Moser et al., 2014) may in part arise from the difficulty of larger scales to qualify as grids. The ratios of the grid spacing between two consecutive modules are not fixed, but vary between 1.27 and 1.4, and are broadly consistent with experimental data.

The clustering indicated by the distribution of grid spacing around certain values can also be revealed if we rank units according to their grid spacing, and then consider the absolute difference of grid spacing between units. This ordered bi‐dimensional map is shown in Figure 3c and clearly exhibits a community structure, where units belonging to the same module have a small difference in grid spacing (white color). Colored corners indicate the limits of each module. Quantitatively, the “discreteness” was defined by (Stensola et al., 2012) as the standard deviation of the statistics given by the collection of unit counts in all bins of the (binned) distribution of grid spacing, for a particular bin width. In agreement to that study, we found a consistent large discreteness value, irrespective of the bin width, clearly separated from a control uniform‐like distribution (not shown).

In addition to grid spacing, the orientation of the hexagonal pattern is essential to define a common representation and, therefore, modularity itself. A common alignment among units can be revealed clearly by looking at autocorrelograms, which are independent of the spatial phase of individual units. The six centers of the hexagonal pattern can be extracted from each autocorrelogram and then plotted across units, as in Figure 3d. To visualize the association between individual representations, the six centers were colored differently according to their position in the plane. In fact, due to symmetry properties of autocorrelograms, only three of those are independent (e.g., those in the upper hemiplane). As seen in the Figure, the centers of the individual hexagonal patterns cluster together, especially for the modules with the smallest grid spacing. This clustering, therefore, should be also reflected in the orientation at the population level. In Figure 3e we show the joint histogram of the grid spacing and orientation of the three independent axes of the grid pattern, where the segregation of the first three modules is clear. In this plane, the similar distribution across the three axis separated roughly by 60° reflects the symmetry of the underlying hexagonal structure, although there are systematic variations that we will consider below. Considering one particular axis or all three, we can observe that the orientation of the grids is, unlike its spacing, strongly correlated across these three modules, though not identical. Furthermore, although collateral connections have a definite range, their influence spreads through correlated activity well beyond this range. This can be inferred from the common orientation developed by all units in the second and third modules, even when individual units are in one of two separate branches of the dorso‐ventral gradient. The fourth module (as in general all units with large spacing), is not only statistically unreliable due to the few units it includes; it also has a poorer aggregation. Both observations are in agreement with the experimental data reported in (Stensola et al., 2012).

As suggested by Figure 3d, individual grid patterns are not exactly hexagonal, and some variability in grid spacing and inter‐axis angles is observed. However, this variability is not random. Axes stretch and shrink producing an ellipse, instead of a circle where the vertices of the hexagonal pattern are supposed to lay on. This distortion can be quantified by fitting best ellipses to the six surrounding fields in the individual autocorrelograms, and comparing the tilt and the eccentricity (or ellipticity) at the population level. This population‐based analysis can be performed by constructing the histogram of the points whose polar coordinates are given by the eccentricity (radius) and the doubled tilt angle (polar angle). As observed in Figure 3f, this “distortion” map exhibits largely a single cluster, indicating that all units tend to produce ellipses oriented in the same direction, irrespective of the module they belong to, with larger distortions (eccentricities) for units exhibiting the largest grid spacing values (not shown). This is also in agreement with some of the experimental data reported in (Stensola et al., 2012), where they have found this coherent distortion but also a bi‐modal structure of the elliptical organization. Because the distortion does not provide information for the modular architecture in our simulations, we rely on spacing and orientation to define modules with minimal arbitrary manipulation. This is achieved with a k‐means clustering selection procedure, with four modules set ad hoc, *k* = 4 (although other values were also considered). As the procedure does not produce a unique deterministic outcome, we run the analysis 300 times and evaluated the performance of each repetition by means of the population‐based mean of the so‐called silhouette coefficient Cs, see (Stensola et al., 2012) for further explanation. Based on the highest mean Cs, the best clustering outcome was selected and the mean properties of each cluster or module, as well as its cardinality, were obtained. A summary is given in Table 1. Properties obtained by the *k*‐means procedure confirm the modular structure already revealed by the analysis based on the different histograms at the population level.

**Table 1:** Properties of the *k* = 4 modules extracted by the *k*‐means procedure

	Module 1	Module 2	Module 3	Module 4
Spacing Axis 1	45.1 ± 2.2 cm	57.1 ± 3.5 cm	74.6 ± 8.9 cm	104.6 ± 15.7 cm
Spacing Axis 2	46.0 ± 3.3 cm	59.8 ± 4.3 cm	77.0 ± 7.3 cm	107.7 ± 12.6 cm
Spacing Axis 3	45.3 ± 3.4 cm	58.7 ± 2.8 cm	75.1 ± 6.7 cm	103.7 ± 8.6 cm
Orientation Axis 1	−3.9 ± 4.6 deg	0.0 ± 11.8 deg	0.3 ± 12.7 deg	6.9 ± 14.0 deg
Orientation Axis 2	52.3 ± 4.1 deg	60.6 ± 11.8 deg	61.6 ± 13.5 deg	71.3 ± 21.2 deg
Orientation Axis 3	−66.7 ± 5.5 deg	−62.5 ± 11.2 deg	−63.3 ± 12.9 deg	−53.3 ± 17.3 deg
Number of units	19	31	36	8

Because all previous results may critically depend on the threshold imposed on the grid score for further consideration of individual units, we analyzed the effects of varying this threshold, which in turn produces smaller/larger datasets. Figure 3g shows the smoothed histograms for different threshold values, whereas Table 2 indicates the number of units and the mean Cs obtained with the (best selected) *k*‐means procedure. Larger values of mean Cs quantifies domains for data points that are better resolved by clusters, with increasingly less degree of overlap between them. A mean Cs of 0.5 may be considered a consistent separation. As noted, the modular structure is reliable, beyond the particular value of the threshold. Naturally, as this value increases, fewer units remain in the dataset but, on the other hand, the quality of the definition of the clusters is enhanced.

**Table 2:** Number of units and mean Cs of the (best) *k*‐means clustering procedure, for datasets produced with different thresholds on the grid score

	Number of units	Mean Cs (*k*‐means)
Threshold = 0.25	258	0.36 ± 0.22
Threshold = 0.50	135	0.40 ± 0.17
Threshold = 0.60	94	0.45 ± 0.20
Threshold = 0.75	56	0.50 ± 0.16

Total population, 600 U.

## 4 DISCUSSION

Our simulations point at the possibility that grid modularity may not be hardwired but may result from a simple self‐organization process at the local network level. Similarly, the adaptation model (Kropff & Treves, 2008) has earlier demonstrated the possibility that grid structure may emerge from self‐organization at the single unit level.

There is a long list of features in the simulated results that could be compared with the experimentally observed modularity. The degree of abstraction of the model from the biophysics and connectivity of real entorhinal networks, and the many simplifications it entails, make a systematic study not so useful. One aspect, however, deserves attention. The variation of the adaptation time constant with distance along the dorso‐ventral axis was designed, in the model, to produce a linear scaling of grid spacing with such distance. Yet this is not what the simulations produce. The different modules are comprised of different number of units, with the fourth one having the least number. Moreover, grid spacing takes values that resemble more a geometric progression (albeit with slightly variable coefficient, from ca. 1.27 to ca. 1.4 between the third and fourth module) than a simple linear one. This is reminiscent of the progression of values, geometric on average, observed experimentally, with mean coefficient just above 1.4 (Stensola et al., 2012). Note however that in our model the geometric coefficient is not imposed to optimize any representational capacity, as in the argument by (Mathis, Herz, & Stemmler, 2012; Mosheiff, Agmon, Moriel, & Burak, 2016; Sanzeni, Balasubramanian, Tiana, & Vergassola, 2016;), as it just emerges from the mechanics of self‐organization.

As to the logarithmic form of the variation of the time constant, it is at odds with the early observation of characteristic resonance timescales (Giocomo, Zilli, Fransén, & Hasselmo, 2007), but in a conservative direction. If we had assumed a linear form, for example, a strong, perhaps exponential increase in spacing towards the ventral end would have been expected a priori. Therefore the simulations suggest that the geometric progression observed may not just reflect the underlying variation in single cell properties, but also network self‐organization effects. Are single cell properties in our adaptation model consistent with experimental evidence? Yoshida, Jochems and Hasselmo (2013) argue that the stronger firing rate adaptation they find in more ventral portions of medial entorhinal cortex layer II contradicts the assumption of the adaptation model. The model does not assume, however, stronger adaptation in more dorsal mEC, but rather a faster recovery from adaptation; and a faster set of characteristic time constants, from AHP duration to (inverse) subthreshold membrane potential oscillation frequency to input resistance, is precisely what Yoshida et al. (2013) measure, providing experimental support for the adaptation model. Their correlation analysis does not have the resolution to clarify the exact trend with which such single cell properties, tentatively associated to the time constant required in our model, may vary along the dorso‐ventral axis, but it appears that the relationship is quite noisy anyway. Note also that in a recent report an inverse progression has been observed in bats (Heys et al., 2016). In this article, the authors performed whole‐cell patch recordings along the dorsal–ventral axis of EC in bats and, surprisingly, found that the sag response properties and the resonance properties recorded in layer II neurons of entorhinal cortex in the Egyptian fruit bat demonstrate an inverse relationship along the dorsal–ventral axis compared with the rat.

Pilly and Grossberg (2014) have produced an account of the emergence of modularity based on “stripe” cells, where essentially by two‐dimensional superposition grid units emerge. For the modules in turn to emerge, they use populations of stripe cells with different a priori spacing, so it remains unclear how distinct modules self‐organize. In this sense, our study helps to clarify possible mechanisms by which modules are produced, highlighting the potential role of collateral interactions during this process.

Finally, one may wonder about the origin of the total number of modules observed in each animal. In this respect, it seems premature to attempt to establish firm relationships with observations in rodents, for at least two reasons. First, both modularity and gridness appear to be elusive and increasingly ill‐defined notions as one approaches the ventral end of entorhinal cortex, suggesting caution in extending there an idealized conceptual model that only really applies to the units with shorter spacing at the other end. Second, observations and considerations arising particularly in the case of bats (Geva‐Sagiv, Las, Yovel, & Ulanovsky, 2015) with their behavioral need for widely divergent length scales, suggest the possibility that grid‐like units may reveal much more flexible regimes, where for example, their spacing is modulated by ongoing behavior, than so far observed in stereotyped experiments in rodents. Such flexibility would open an entirely new perspective on the emergence and the role not just of modules but also of grid cells themselves, further highlighting the urgency of understanding their self‐organization.

## ORCID

*Alessandro Treves* http://orcid.org/0000-0001-7246-5673

## APPENDIX 1

The model has been described in detail by Si et al. (2012). Briefly, each unit in the conjunctive cell layer has a firing rate given by

(A1) $$\Psi_{i}^{t}=\Psi_{\text{sat}}\;\tan^{-1}[g^{t}(\alpha_{i}^{t}-\mu^{t})]\;\Theta \;(\alpha_{i}^{t}-\mu^{t})$$

where Ψ_sat_ = 2/π is the maximum firing rate (in arbitrary units), Θ(.) is the Heaviside function, and *g* ^t^ and μ^t^ are the gain and the threshold of the nonlinearity, which are dynamically adjusted during simulations in order to satisfy conditions on the mean activity, *a* = Σ_i_ Ψ_i_/*N*_mEC_ = 0.1, and the sparsity, *s* = (Σ_i_ Ψ_i_)^2^/[*N*_mEC_ Σ_i_ (Ψ_i_)^2^] = 0.3, where the sum runs over units in the conjunctive layer. The variable α_i_ represents a filtered version of an input signal h_i_,

(A2) $$\alpha_{i}^{t}=\alpha_{i}^{t-1}+b_{1}(h_{i}^{t-1}-\beta_{i}^{t-1}-\alpha_{i}^{t-1})$$

adapted by the dynamical threshold

(A3) $$\beta_{i}^{t}=\beta_{i}^{t-1}+b_{2}(h_{i}^{t-1}-\beta_{i}^{t-1})$$

where β_*i*_ has a slower dynamics than *α_i_*, *b*_2_ = b_1_/3, with b_1_ varying according to the dorso‐ventral position of the specific unit *i* (see main text). The overall input, at time *t*, of a given conjunctive unit is

(A4) $$h_{i}^{t}=f_{\theta_{i}}(\phi^{t})\left(\sum_{j}W_{ij}^{t-1}r_{j}^{t}+\rho \sum_{k}{\hat{W}}_{ik}\Psi_{k}^{t-\tau }\right)$$

where the first term in the brackets determines the feedforward contribution from place units, located one synapse downstream when looking at the feedback branch in the CA1‐mEC connectivity layout, and the second term the contribution resulting from collateral interactions. The factor *f*_θi_(ϕ) multiplying both contributions is the head direction modulation characterizing each conjunctive unit, specified by its preferred direction θ_i_ and the inverse of the tuning width ν according to

(A5) $$f_{\theta }(\phi )=c+(1-c)\;e^{\nu [\cos(\theta -\phi )-1]}$$

with a basal modulation, *c* = 0.2. As mentioned in the main text, the inverse tuning width ν varies along the dorso‐ventral axis, whereas each individual θ_i_ is assigned by randomly sampling the 2π range when establishing unit properties before the simulation starts.

The feedforward contribution in Equation A4 is determined by the activity of “place units” (see Kropff & Treves, 2008, for a discussion of this simplification), each one approximated by a Gaussian function centered in a pre‐determined preferred firing location **x**_j0_, with a width σ_p_ = 5 cm, according to

(A6) $$r_{j}^{t}=\exp\left(-\frac{{\left|x^{t}-x_{jo}\right|}^{2}}{2\sigma_{p}^{2}}\right).$$

This small width of place fields ensures that small grid spacing values may be attained at the dorsal end. In our model this width is constant, unlike the variable scale found also for place cells along the dorso‐ventral axis in the hippocampus, which may lead to the relatively small fields developed by grid units at larger grid scales, see Figure 1b. However, representing the complete environment at different scales at the level of the inputs is computationally prohibitive and, as we have indicated, it is not critical for the development of the modular structure, although it may influence the stability and the number of grid units in the modules expressing large scales.

The preferred firing locations of place units are arranged in the 2D circular environment with a radial structure, in which units are displayed on successive rings separated by ∼3.3 cm with a distance between units along the ring circumference of also ∼3.3 cm. As observed in Equation A4, each place unit contributes to the input of a given conjunctive unit with a weight W_ij_, which is not static but changes dynamically according to a Hebbian learning rule,

(A7) $$\begin{array}{c} W_{ij}^{t}={\left[W_{ij}^{t-1}+\in \left(\Psi_{i}^{t}r_{j}^{t}-{\bar{\Psi }}_{i}^{t-1}{\bar{r}}_{j}^{t-1}\right)\right]}^{+}\;\;\; \end{array}$$

where [.]^+^ indicates the threshold function ([*x*]^+^ = 0 if *x* < 0, *x* otherwise) and ɛ is the learning rate, here evolving in a kind of simulated annealing from a fast to a slow learning process, ɛ_initial_ = 0.005 and ɛ_final_ = 0.001 reached at 3/4 of the total simulation time, and thereafter constant. Over‐lined symbols are running time‐dependent averages of corresponding variables. After updating, all weights are normalized according to Σ_j_ (W_ij_)^2^ = 1, mimicking a homeostatic control of synaptic processes. To encourage each unit to join one module, we set the simulation time at about five times the timescale needed to produce reliable gridness and alignment (see Si & Treves, 2013); i.e. here, total simulation time = 10^6^ s.

The contribution of collateral interactions in Equation A4 is determined by the reverberated activity of all other conjunctive units, Ψ_k_, evaluated after a delay τ = 25 time steps (corresponding to ∼250 ms), weighted by *Ŵ*_ik_. These collateral connections are formulated with the prescription given in (Si et al., 2012), which mimics a long learning process matching individual conjunctive properties, here restricted to a local interaction in the arrangement considered for the heterogeneous population (see Figure 2). In detail, those connections in the range of interaction (|*i* − *j*| ≤ *N*_lat_ = 100) are set as

(A8) $${\hat{W}}_{ik}={\left[f_{\theta_{k}}(\phi_{ki})f_{\theta_{i}}(\phi_{ki})\;\exp\;\left(-\frac{d_{ki}^{2}}{2\sigma_{f}^{2}}\right)-\kappa \right]}^{+}$$

whereas those outside this range are zero. In this equation, the head direction selectivity of both pre‐ and post‐synaptic units modulates weight strength, such that it is maximal when their tuning is aligned. In addition, to mimic learning in coactive conjunctive units, each unit is assigned a spatial selectivity (a place field), in a location randomly selected in the environment (Kropff & Treves, 2008). The “coactivity” relevant to our synaptic modification rule is the simultaneous activation of the postsynaptic and the reverberated activity of the presynaptic units. The reverberated activity of the presynaptic unit is assumed to be the activity of the unit a number of time steps before (a delayed internal reverberation, here 25 time steps), when the simulated rat is in a different position from the current one. The coactivation associates therefore two positions a distance apart, the distance travelled during reverberation (speed x reverberatory time = 10 cm, with our parameters), here labeled *d*_ki_ in Equation A8, see (Si et al., 2012) for further details. The direction joining the place fields of pre‐ and post‐synaptic units, ϕ_ki_, defines the argument for the head direction alignment. A diluted connectivity is imposed via the parameter κ = 0.05 and the threshold function [.]^+^. As expressed in Equation A8, this parameter controls which argument of the threshold function will become negative and therefore, which weights vanish. Thus, only units aligned and close in spatial selectivity between reverberated pre‐ and post‐synaptic activity will interact through collateral connections. In other words, this procedure produces a sparse connectivity where a fraction of about 1/10 connections are effectively instantiated. Importantly, these connections are allowed within the neighborhood of any target unit, |*i* − *k*| = 1, …, *N*_lat_ (see main text). The relative contribution of collateral interactions to feedforward processing of “place unit” input is weighted by a factor ρ, see Equation A4, which here is taken to vary along the dorso‐ventral axis (see main text and Figure 2).

The model described above is driven by the random exploration of a virtual agent, constrained in a circular environment of 2 m diameter and moving with a constant speed of 40 cm s^−1^. At each time step, the running direction ϕ is randomly updated from a Gaussian distribution with zero mean and standard deviation 0.2 radians, and then used to compute the coordinates of the future position, considering the speed constant. If this position lies beyond the limits of the circular environment, the trajectory is reflected. With this procedure, the arena is covered homogeneously. In a more realistic situation, where the agent covers the environment with a variable running speed, it is the mean velocity that determines the grid spacing, as the slow Hebbian learning process averages out fluctuations (Kropff & Treves, 2008).

The programming code of the model (written in C) and the population results analyzed here are available in a public repository: “ModelDB, accession code 231392”, http://senselab.med.yale.edu/ModelDB/showModel.cshtml?model=231392.
